# Bioengineered Zinc Oxide Nanoparticle-Loaded Hydrogel for Combinative Treatment of Spinal Cord Transection

**DOI:** 10.3389/fbioe.2021.796361

**Published:** 2022-01-13

**Authors:** Sen Lin, Hao-sen Zhao, Chang Xu, Zi-peng Zhou, Da-hao Wang, Shu-rui Chen, Xi-fan Mei

**Affiliations:** ^1^ Department of Orthopedic, First Affiliated Hospital of Jinzhou Medical University, Jinzhou, China; ^2^ Department of Endocrinology, First Affiliated Hospital of Jinzhou Medical University, Jinzhou, China

**Keywords:** zinc oxide nanoparticles, hydrogel, spinal cord injury, immunomodulation, neuroinflammation

## Abstract

Spinal cord injury (SCI) is one of the most destructive diseases. The neuroinflammation microenvironment needs comprehensive mitigation of damages. Thus, regulation of local, microenvironment drugs could be a potential effective treatment. However, clinical studies on SCI with common treatment have reported it to cause systemic toxicity and side effects. Zinc oxide nanoparticles (ZnONPs) have been widely reported to have satisfying anti-inflammation function. Furthermore, green synthesis procedures can improve the capability and possible utilization of ZnONPs. However, the efficient administration and underlying mechanism of ZnONPs in SCI treatment remain unclear. Herein, an innovative approach was built by utilizing ZnONPs loaded in a skeletal muscle-derived adhesive hydrogel (ZnONPs-Gel). Different from the systemic application of ZnONPs, the local administration of ZnONPs-Gel offered the ZnONPs-loaded extracellular matrix with beneficial biocompatibility to the injured spinal cord, thereby promoting effective function recovery. Mechanistically, the ZnONPs-Gel treatment not only markedly reduced ROS production but also decreased apoptosis in the injured spinal cord. Therefore, the strategy based on local administration of the ZnONPs-Gel in the early stage of SCI may be an effective therapeutic treatment.

## Introduction

Spinal cord injury (SCI) is a destructive disease of the central nervous system (CNS) (McDonald and Sadowsky, 2002), accompanied by motor and/or sensory dysfunctions. Secondary injury followed by primary trauma in the development of SCI includes impairment of the extracellular matrix (ECM), the activation of reactive oxidative stress (ROS), and neuroinflammation (Fawcett, 2015; Ahuja et al., 2017). Several methods have been used clinically such as spinal canal decompression intervention and high-dose corticosteroid (McDonald and Sadowsky, 2002). However, these approaches cause severe side effects, which is a clinically serious problem in patients with SCI that has not yet been resolved (McDonald and Sadowsky, 2002). Therefore, comprehensive strategies of the injured spinal cord are critical to SCI treatment.

Nanoparticles (NPs) are characterized as substances with a size of 1–100 nm (Boraschi et al., 2017). It has been indicated that the application of NPs have anti-inflammation and anticancer function (Baetke et al., 2015; Dadfar et al., 2019). Zinc, as an essential element, is involved in various metabolic processes *in vivo* (Frederickson et al., 2005). The pathological imbalance of the zinc level causes various disorders of the CNS, such as epilepsy and dementia (Pochwat et al., 2015). Zinc plays an important role in the formation and maturation of fetal CNS (Frederickson et al., 2005). As a common zinc-contained nanomaterial, zinc oxide nanoparticles (ZnONPs) have been reported to be applied in biological research because of their low toxicity, biocompatibility, and bioactivity (Basnet et al., 2018). Therefore, local application of ZnONPs will ameliorate the inflammatory microenvironments and therefore attenuate injured spinal cord dysfunction.

Hydrogel has been suggested as a promising therapy for trauma-related diseases such as SCI and wound healing (Zhao et al., 2017; Koffler et al., 2019). Previous studies have demonstrated that skeletal muscle-derived hydrogel has sufficient adhesion and histocompatibility (Ungerleider et al., 2015; Hernandez et al., 2020). We presented a novel promising treatment for the local delivery of ZnONPs in injured spinal cord based on skeletal muscle-derived hyaluronic acid (HA) hydrogel (ZnONPs-Gel). In this study, the implantation of ZnONPs-Gel effectively recovered the hindlimb motor function in SCI mice *via* regulating the focus microenvironment and suppressing inflammation and ROS. This study developed an innovative strategy for the local delivery of ZnONPs for SCI treatment.

## Materials and Methods

### Culture of Primary Bone Marrow Mesenchymal Stem Cells

According to a previous study (Li et al., 2016), BALB/C mice (4–5 weeks) were killed after being anesthetized and soaked in 75% ethanol for 5 min. Bilateral femurs were separated on a sterile operation platform. The femurs with the muscle tissues around being removed were soaked in the dish containing saline. The syringe took an appropriate amount of culture medium containing 10% FBS to flush the bone marrow into the culture bottle. The bone marrow cell suspension was blown repeatedly to make a single-cell suspension. The cell suspension was centrifuged at 1,000 rpm for 5 min, and then the cells were collected. The cells were cultured at 37°C under 5% CO_2_.

### Isolation and Characterization of Biosynthetic ZnONPs *In Vitro*

According to previous studies (Ogunyemi et al., 2019), the produced ZnONPs were isolated from the culture supernatant of BMSCs. The medium was centrifuged for 5 min at 900  ×  g followed by centrifuging for 1 h at 10,000  ×  g to remove cell debris, and the supernatant was filtered with a 0.2-μm pore filter. After that, the samples were centrifuged 30 min at 4°C and 400  ×  g, followed by passing through a CL-2B column. Then, the filtrate was used with a freeze-dryer. The characterizations of ZnONPs were performed by transmission electron microscopy (TEM), energy-dispersive X-ray spectroscopy (EDS), and X-ray diffraction (XRD).

### Fabrication of ZnONPs-Loaded Hydrogels

For a typical fabrication of tissue-specific hydrogels (Ungerleider et al., 2015), 20–35 mg of skeletal muscle was added into 800 ml of 1% wt/vol sodium dodecyl sulfate (SDS) solution and stirred at 125 rpm for 2 h. After being rinsed with ultrapure water, tissue was added to a certain volume of fresh 1% SDS solution and spun at 125 rpm for 24 h. An aqueous solution of isopropyl alcohol (400 ml) was added slowly to the mixture, and the stirring was continued for 12 h. Afterward, ZnONPs were added to the samples, and ECM was used with a freeze-dryer. Frozen ECM is lyophilized and ground to generate particles for subsequent protease digestion. Fresh pepsin was dissolved in 0.1 M HCl at a rate of 1 mg/ml by shaking for 5–10 min. While the pepsin is shaking, approximately 20–30 mg of ground ECM was added to a 20-ml scintillation vial with a small stir bar. The closed vial was then placed on a stirring plate (60–120 rpm) at room temperature for 48 h. To ensure that the entire material is digested in the pepsin solution, a spatula was used to gently scrape off the material on the side of the vial once or twice during the 48-h digestion process. After 48 h, the liquid ECM was titrated to pH 7.4 PBS, and the final concentration was 6 mg ECM/ml.

### SCI Model and Treatments

Male BALB/C mice (weighing 22–28 g) were used for this study. Rats were fed in a controlled place with standard rodents. Animals were stayed at 22 ± 1°C with a half-day light, half-day cycle. The study complied with the Animal Research: Reporting of *In Vivo* Experiments (ARRIVE) guidelines and obtained permission by the Jinzhou Medical University Review Board for the care of animals. Complete contusion SCI mice were prepared as previously described. An impounder (2 mm diameter, 10 g) was fallen on the T9–T10 spinal cord from a 2.5-cm height to form a spinal cord moderate contusion. The bladder was massaged twice a day until bladder function recovered normally. The blank group was transplanted with hydrogel. The ZnONPs-Gel group was transplanted with hydrogel-loaded ZnONPs.

### Behavioral Assessment

The behavioral assessment was tested by behavioral analysis using the Basso Mouse Scale (BMS) open-field locomotor test (Basso et al., 1995). Double-blind assessment was used at 0, 1, 3, 7, 14, 21, and 28 days post-injury. BMS scores range from 0 to 9 points. The 0 point indicates complete paralysis, and 9 points indicates normal function. The average scores were calculated in accordance with the grading standard in locomotion recovery after SCI.

### RT-qPCR

The injured spinal cord tissue was collected from the time point for the experiment of RT-qPCR. The relative expression levels of the target genes were normalized to those of the housekeeping gene ribosomal protein S18 (RPS18), and the target genes from the experimental group were compared with the corresponding target genes from the control group using the (1 + e)^−ΔΔCT^ method. The following oligonucleotide primers are listed in Table 1.

**TABLE 1:** Primer sequences used for quantitative real-time PCR.

Gene	Forward primer (5′ to 3′)	Reverse primer (5′ to 3′)
iNOS	TTT​GCC​AAT​TCA​TTA​CTT​CCA	ATC​ACA​CCG​CCT​CCT​GAT​TCC
Arg-1	CTC​CAA​GCC​AAA​GTC​CTT​AGA​G	AGG​AGC​TGT​CAT​TAG​GGA​CAT​C
RPS18	GCA​ATT​ATT​CCC​CAT​GAA​G	GGC​CTC​ACT​AAA​CCA​TCC​AA

### Western Blot

At 7 days post-operation, the injured spinal cord (1 cm from the center of the injury point) was removed. Tissues were chopped and then dissolved in RIPA lysis buffer. The same amount of protein samples was added into polyacrylamide gels. The samples were added to SDS-PAGE and transferred to a membrane, then blocked with 1% BSA in TBST at room temperature for 2 h. Then, the membranes were immersed with the primary antibodies at 4°C overnight. On the second day, membranes were incubated with the secondary antibodies at room temperature for 2 h. The membranes were imaged by using the ChemiDoc-It™ TS2 Imager, and relative optical density was analyzed by ImageJ2x software.

### Histological Staining and Immunofluorescence Staining

Mice were anesthetized with urethane (20%, 5 ml/kg) after operation. A 5-mm segment of the spinal cord including the injury lesion was taken. The segments were soaked in 4% paraformaldehyde for 3 days and added to 30% sucrose in 4% paraformaldehyde for 3 days. For HE staining, frozen sections were dried at room temperature for 30 min and immersed into hematoxylin for 6 min, the slides were sluiced in running water for 10 s. The sections were differentiated in HCl/95% alcohol (1:50) solution for 5 s. After washing with running water for 25 min, the slides were re-stained with eosin and then fixed with neutral balsam after dehydration *via* 75% alcohol, 95% alcohol, and 100% alcohol and transparency with xylene. For immunofluorescent analysis, sections were blocked 5% normal goat serum for 1 h and incubated overnight at 4°C with primary antibodies. Next day, the tissues were rewashed with PBS and incubated with Alexa Fluor-488 or Alexa Fluor-568 at room temperature for 2 h. The nucleus was dyed with DAPI solution (1:1,000).

### ROS Activity Assay

Superoxide dismutase (SOD) and glutathione (GSH) activity in the spinal cord tissue were measured using assay kits (Jiancheng, Nanjing, China) according to the manufacturer’s instructions (Qin et al., 2019).

### Statistical Analysis

Data represented as mean ± SD and analyzed by SPSS 23.0. Student’s t-test and one-way ANOVA were detected the data of two groups and more groups. In addition, the BMS score was analyzed using the Mann–Whitney U-test. Differences were considered statistically significant with a value of *p* < 0.05.

## Results and Discussion

### Characterization of ZnONPs and ZnONPs-Gel

In the synthesis of nanomaterials, the use of biological components has always been the better choice of environmentally friendly methods known as green synthesis. In addition to environmental and ecological benefits, green synthesis has proven to be very useful in controlling the required size and shape. Various studies have shown that the synthesis of animal extracts is more compatible than that of other organisms, so it is more suitable for large-scale synthesis of green NPs (Hussain et al., 2016).

**FIGURE 1:**
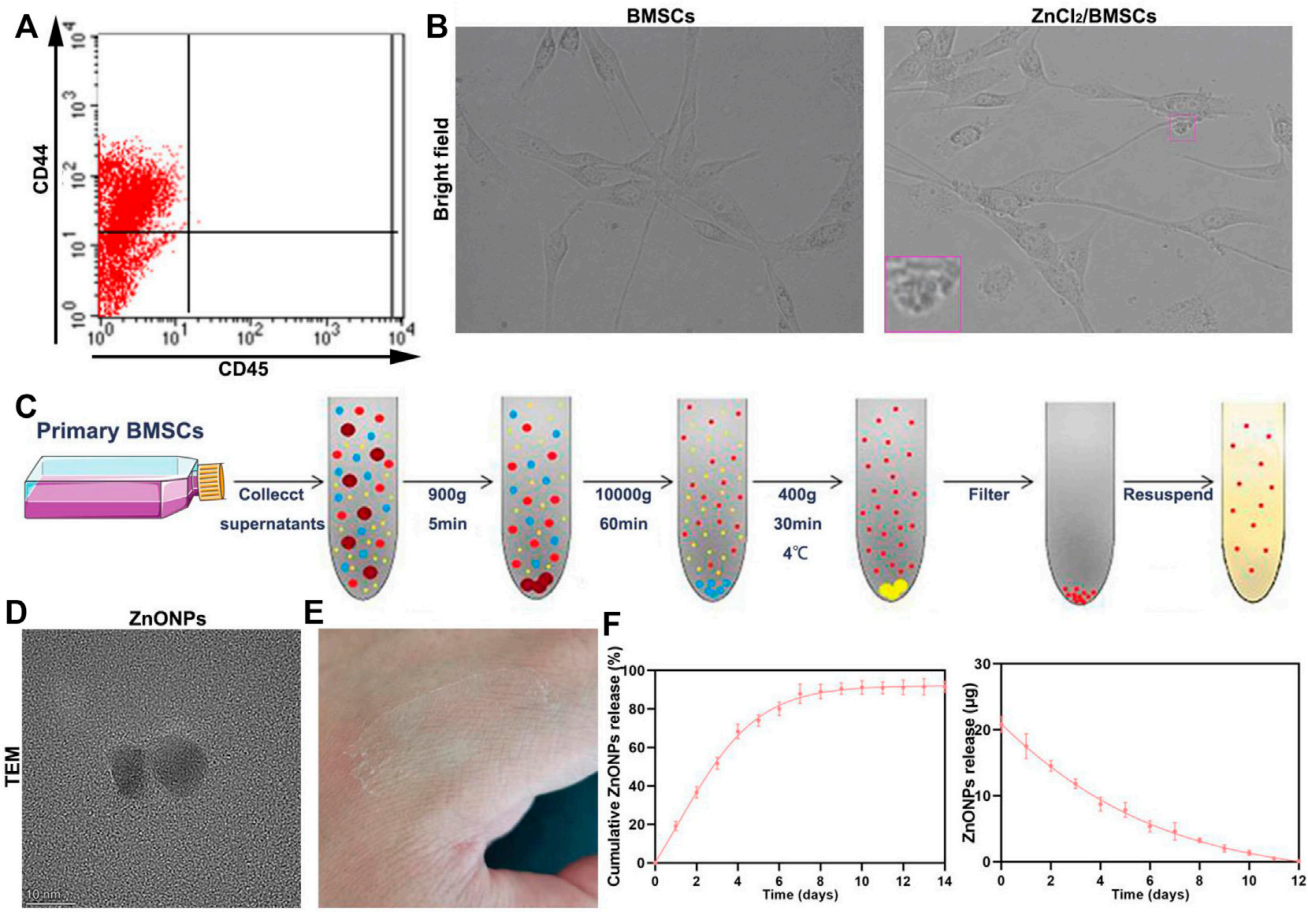
Fabrication characterization of ZnONP-loaded hydrogels. Flow cytometry of primary BMSCs **(A)**. Representative images of BMSCs **(B)**. Scheme of steps for extracting BMSCs **(D)**. Representative image of ZnONPs **(D)**. Representative image of ZnONP-loaded hydrogel solidification **(E)**. Representative quantifications of ZnONP release **(F–G)** in ZnONPs-Gel. Data are mean ± SD (*n* = 3).

Flow analysis showed that BMSCs had high expression levels of CD44 and low expression of CD45 (Figure 1A) (Soleimani and Nadri, 2009). The cultured BMSCs were fibroblast-like cells, and the BMSCs formed homogenous colonies. Most of the BMSCs had clear cellular boundaries and administration of zinc chloride (ZnCl_2_) did not disturb the cells’ form (Figure 1B). ZnONPs were isolated from BMSC culture supernatant and purified by chromatography and ultracentrifugation. The purified ZnONPs showed spherical morphology. The result of TEM indicated that the sizes of the purified ZnONPs ranged from 10 to 50 nm (Figure 1D). The viability of BMSCs was investigated in the absence and presence of ZnONPs based on the MTT experiments (Supplementary Figure S1). After BMSCs were treated 24 h, the cell viabilities were near 100% with ZnONPs, at various concentrations from 0 to 30 μg (exceeded the maximum load of ZnONPs-Gel). Moreover, ZnONPs decreased the ROS expression after being treated with H_2_O_2_ (Supplementary Figure S2). The result showed that green production of ZnONPs significantly reduced cell toxicity. ZnONPs were released continuously from gel for about 14 days, and more than 92% of ZnONPs was finally released (Figures 1E,F). Furthermore, we measured characterizations of ZnONPs and ZnONPs-Gel (Supplementary Figures S3, S4). Therefore, the retention and slow release of ZnONPs from skeletal muscle-derived hydrogel were demonstrated *in vitro*. These data suggested that the adhesion and retention of ZnONPs in gels (ZnONPs-Gel) had possible application prospects in effective delivery.

### Neuroprotective Effect of ZnONPs-Gel *In Vivo*

**FIGURE 2:**
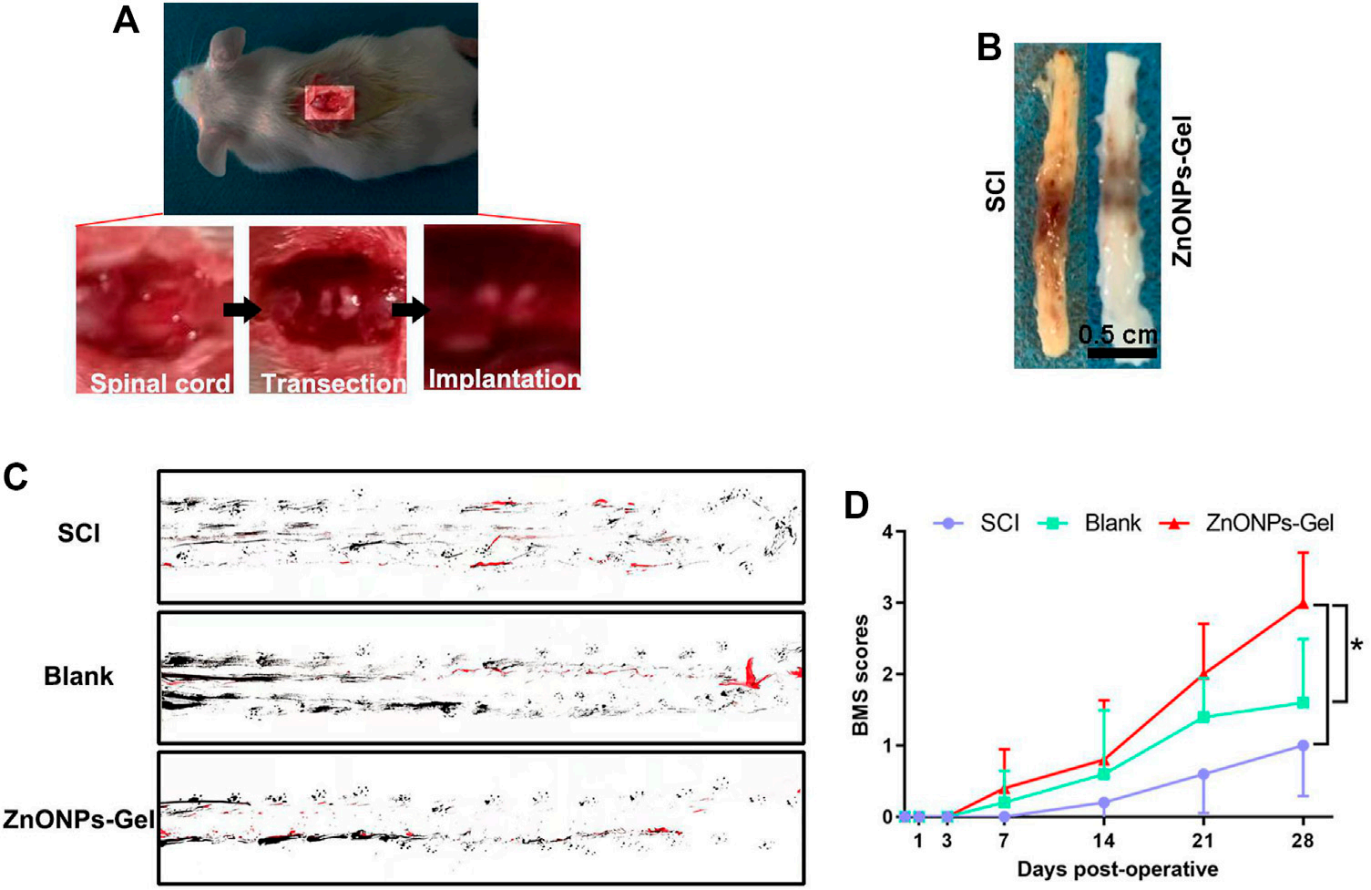
ZnONPs-Gel recovered motor function on days 28 after SCI. Model of injured spinal cord **(A)**. Representative images of the injured spinal cord in SCI and ZnONPs-Gel groups **(B)**. Representative images of footprint analysis in the SCI, blank, and ZnONPs-Gel groups **(C)**. Representative quantification of BMS scores in SCI, blank, and ZnONPs-Gel groups **(D)**. Data are mean ± SD (*n* = 3).

Gel treatment (blank group) and ZnONPs-Gel treatment (ZnONPs-Gel group) were performed to evaluate the effect of implanted ZnONPs in a severe long-span spinal cord transection model in mice (Figures 2A,B). Mice received only PBS after transection serving as the control group (SCI group). The animals from the SCI group were almost completely paralyzed lasting 28 days after SCI, while the implantation of ZnONPs-Gel significantly recovered motor function (Figures 2C,D). The findings demonstrated that ZnONPs-Gel administration had a significant effectiveness on nerve recovery.

**FIGURE 3:**
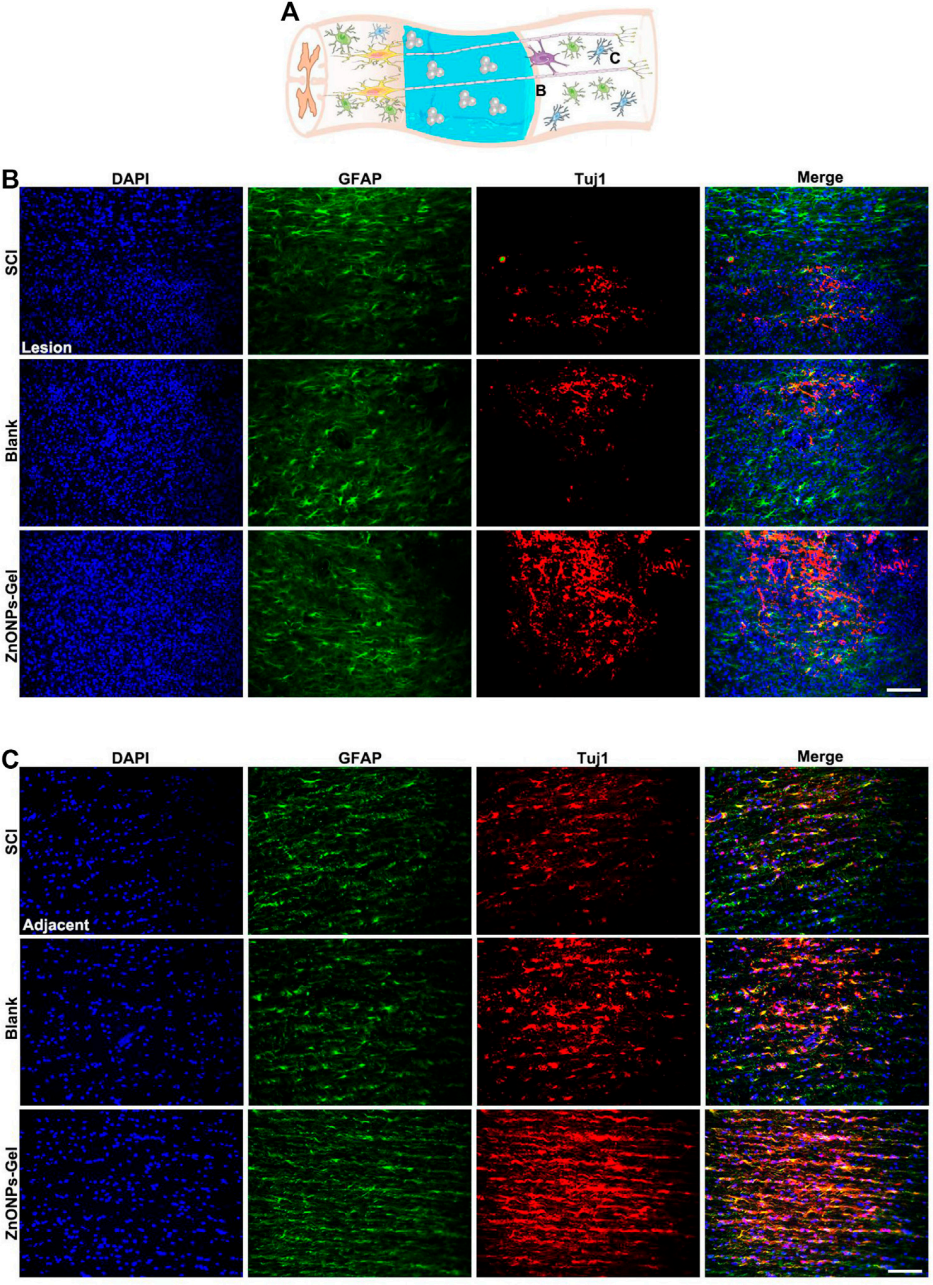
ZnONPs-Gel improved injured the spinal cord on days 28 after SCI. Model of injured spinal cord **(A)**. Representative images showing Tuj1 (red) and glial fibrillary acidic protein (GFAP, green) staining in the lesion **(B)** and the adjacent **(C)** of SCI, blank, and ZnONPs-Gel groups. Scale bar = 100 μm. Data are mean ± SD (*n* = 3).

**FIGURE 4:**
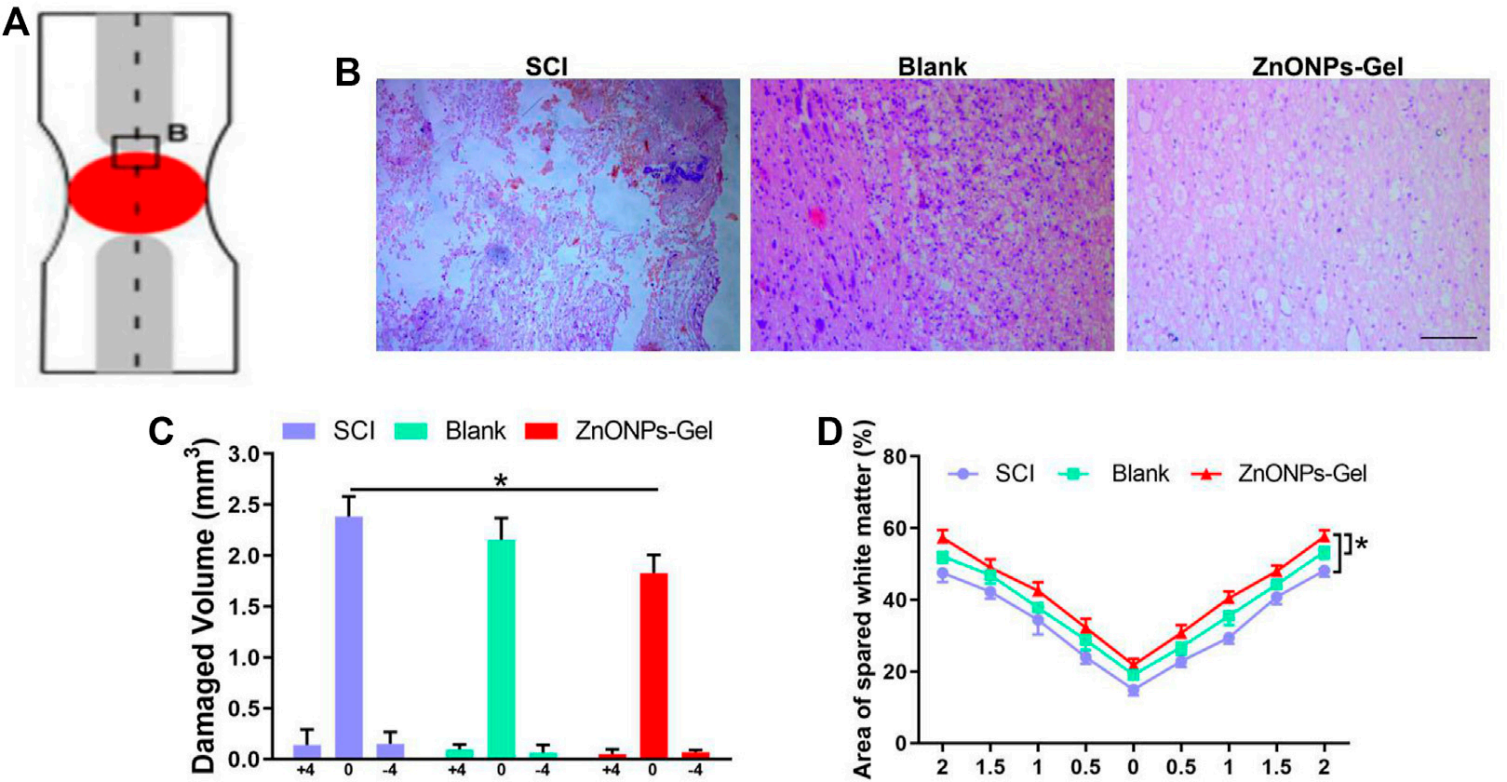
ZnONPs-Gel promoted injured spinal cord tissue repair after SCI. Model of injured spinal cord **(A)**. HE staining **(B)** and quantification of damaged volume **(C)** and area of spared white matter **(D)**. Scale bar = 100 μm. Data are mean ± SD (*n* = 3); *significant difference compared to the SCI group.

Moreover, to investigate the tissue recovery of implanted ZnONPs-Gel, assessment was performed on day 28 after injury *via* immunofluorescence double staining (Figure 3A and Supplementary Figure S5) and HE staining (Figure 4). The distributions of Tuj1 and glial fibrillary acidic protein (GFAP) astrocytes have been reported to represent the degree of nerve tissue recovery. We found that in the blank group, GFAP-positive cells gathered in the margin of the cavity, with few Tuj1 (Figure 3B). Compared with the blank group and SCI group, the double-positive stainings of Tuj1 and GFAP cells in the ZnONPs-Gel group were significantly increased (Figure 3B). As shown in Figure 3C, the double-positive stainings of Tuj1 and GFAP cells in the ZnONPs-Gel group were not different as compared to the blank and SCI groups. The results indicated that the injured spinal cord tissue repaired by ZnONPs-Gel exhibited a higher expression of Tuj1 in different segments, accompanied by fewer astrocytes. On day 28 after implanting ZnONPs-Gel, the cavity was less than that in the SCI and blank groups. Four weeks after injury, a dramatic tissue loss on the injured spinal cord was observed, reflecting that ZnONPs-Gel significantly decreased the lesion volume (Figure 4 and Supplementary Figure S5). Besides the long-term effect of ZnONPs-Gel on SCI which was investigated by a survival curve (Supplementary Figure S6), we found that ZnONPs-Gel promoted long-term SCI recovery and mouse survival. Consistent with Figure 2, these data suggested the important role of ZnONPs in the implantation of nerve repair with hydrogel and supported the effective delivery of ZnONPs with the implantation of ZnONPs-Gel.

### Anti-ROS Effect of ZnONPs-Gel *In Vivo*

**FIGURE 5:**
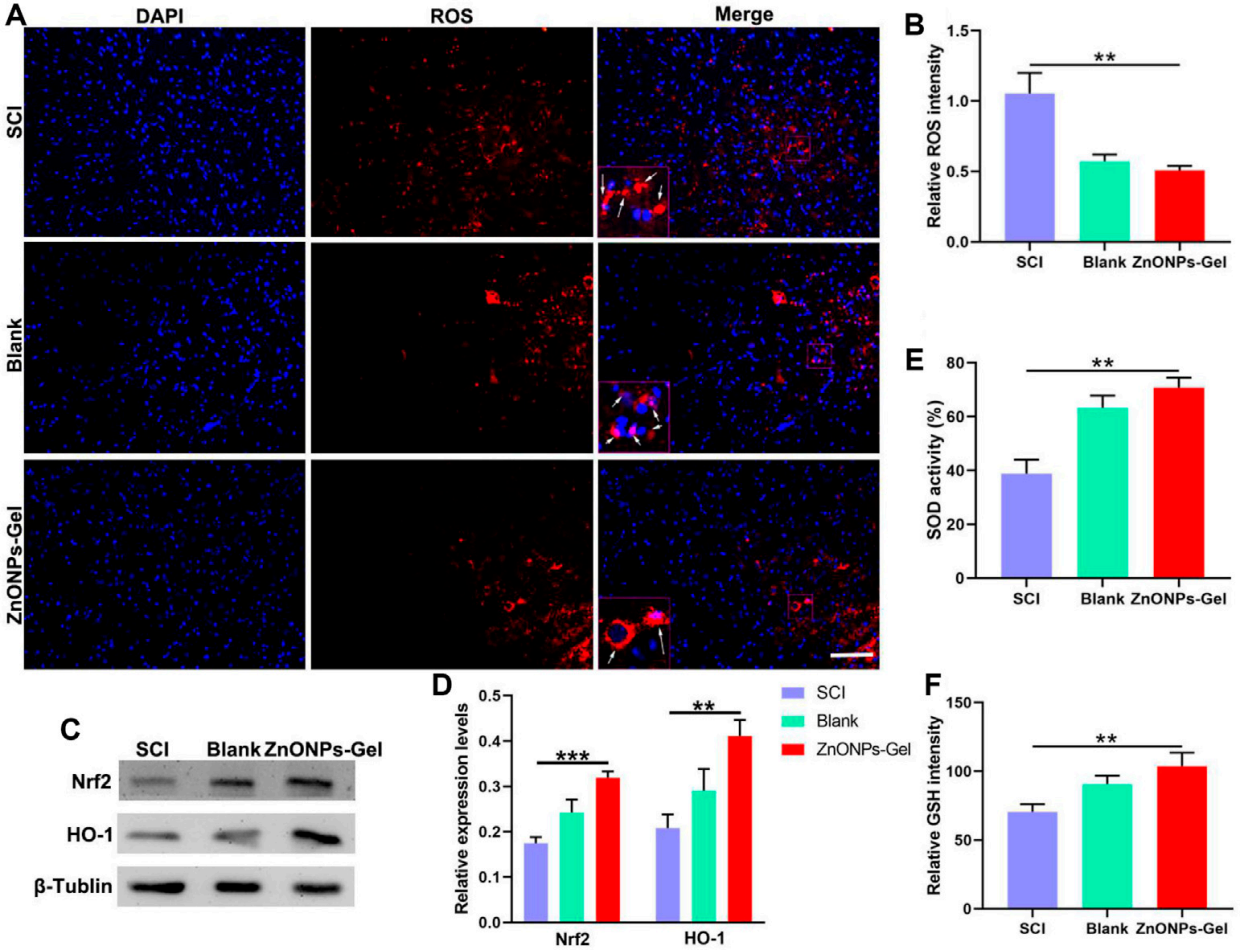
ZnONPs-Gel inhibited injured spinal cord oxidation on days 28 after SCI. Representative images **(A)** and quantification **(B)** showing DAPI (blue) and ROS (red) staining in the lesion of SCI, blank, and ZnONPs-Gel groups. Representative images **(C)** and quantification **(D)** of the expression of Nrf2 and HO-1 in the spinal cord of Sham, SCI, blank, and ZnONPs-Gel groups. Representative quantification showing SOD activity **(E)** and GSH **(F)** assays in the injured spinal cord of SCI, blank, and ZnONPs-Gel groups. Scale bar = 100 μm. Data are mean ± SD (*n* = 3).

SCI resulted in the increase in ROS expression; the activation of ROS caused secondary injury in the acute development of SCI (Shadel and Horvath, 2015). After being treated with ZnONPs-Gel on day 28, the antioxidant markers of SOD, GSH, Nrf2, and HO-1 were detected by ELISA and Western blot. According to Figures 5A,B, ZnONPs-Gel effectively downregulated ROS intensity as compared to the SCI and blank groups. Moreover, implantation of ZnONPs-Gel extended the production of SOD, GSH, Nrf2, and HO-1 in the injured spinal cord tissue (Figures 5C–E). The quantitative analysis of the results showed a significant difference between the ZnONPs-Gel group and SCI group. These findings demonstrated the antioxidant role of ZnONPs-Gel in the treatment of nerve repair after SCI.

### Anti-Inflammation Effect of ZnONPs-Gel *In Vivo*

**FIGURE 6:**
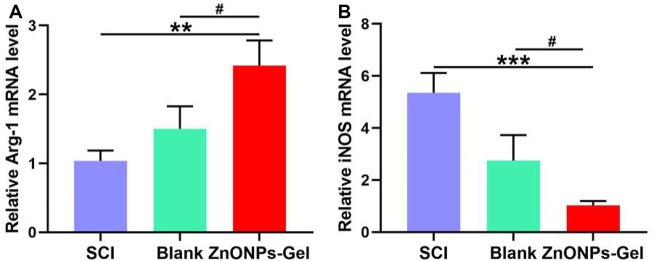
ZnONPs-Gel-polarized M2 markers after SCI. Quantification of expression of M1: iNOS **(A)** or M2 markers: Arg-1 **(B)** in the injured spinal cord of SCI group, blank group, or ZnONPs-Gel group at 7 days during the acute course of SCI. Data are mean ± SD (*n* = 3); *significant difference compared to SCI group (*p < 0.05; **p < 0.01; ***p < 0.001); #, compared to the blank group (#*p* < 0.05).

**FIGURE 7:**
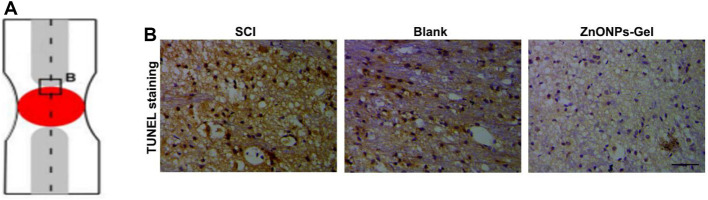
ZnONPs-Gel decreased injured spinal cord tissue apoptosis after SCI. Model of injured spinal cord **(A)**. TUNEL staining **(B)** in the injured spinal cord of SCI group, blank group, or ZnONPs-Gel group at 7 days during the acute course of SCI. Scale bar = 100 μm.

Inducible nitric oxide synthase (iNOS), as a proinflammatory messenger molecule, aggravates the progress of neuroinflammation. Arginase-1 (Arg-1) has been indicated to promote nerve recovery (Hickman et al., 2018). To further extend the neuroprotective effects of ZnONPs-Gel on the hyperinflammation microenvironment after SCI, we detected the mRNA levels of iNOS and Arg-1 by quantitative real-time PCR (RT-qPCR). At 7 days after implantation of ZnONPs-Gel, the expression of iNOS decreased significantly, accompanied by the expression of Arg-1 which increased significantly (Figure 6). These results indicated that ZnONPs-Gel implantation has anti-inflammatory effects in the acute phase of SCI. In addition, the data of TUNEL staining revealed a decrease in nerve death after therapy of ZnONPs-Gel (Figure 7). These findings offered evidence for the underlying mechanism of spinal cord repair by ZnONPs-Gel.

### Immune Response of the Administration of ZnONPs-Gel *In Vivo*

**FIGURE 8:**
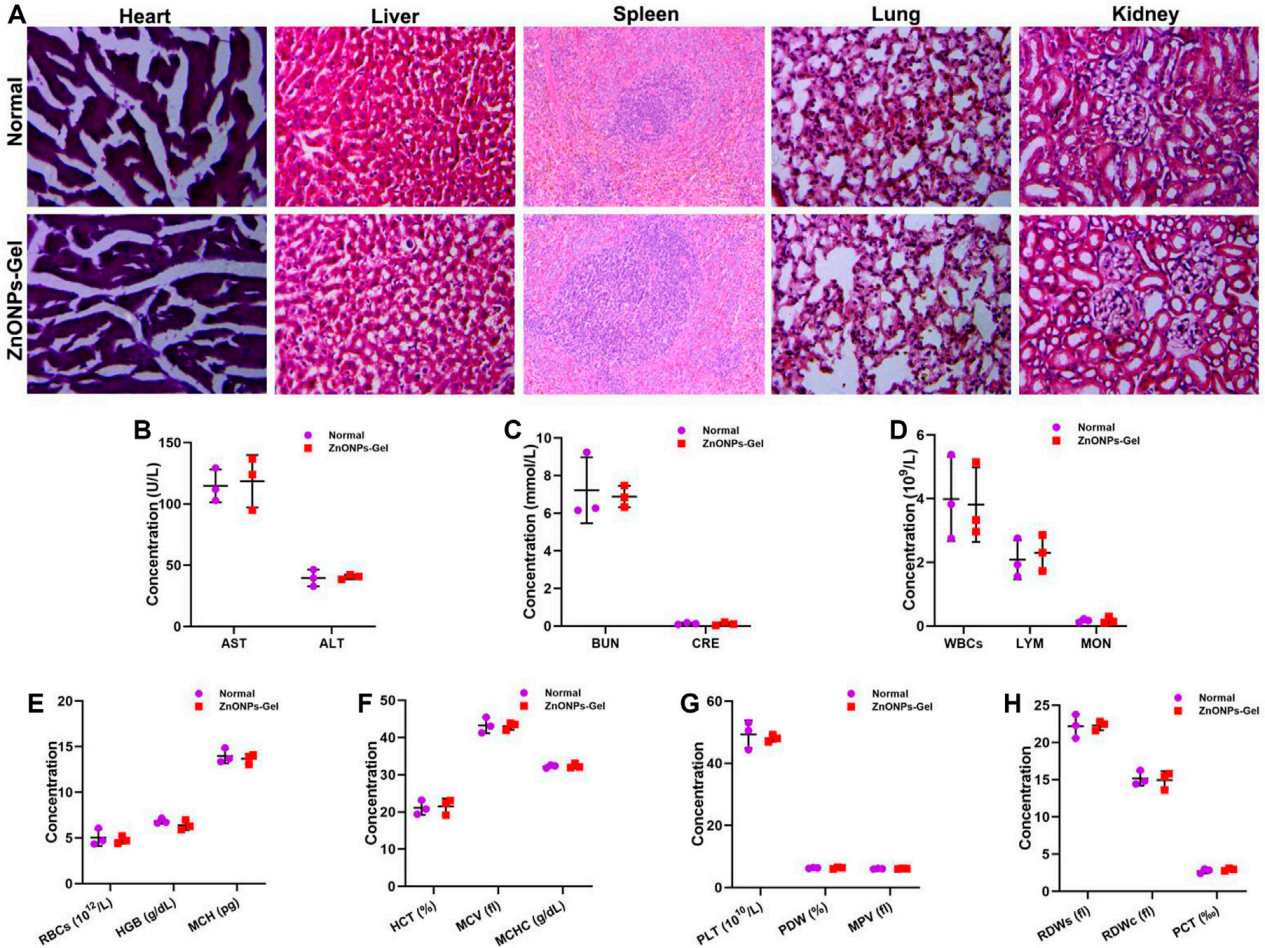
Implantation of ZnONPs-Gel had biological safety in SCI mice. HE staining of heart, liver, spleen, lung, and kidney in normal mice and ZnONPs-Gel-implanted mice **(A)**. Representative quantification of the AST **(B)**, ALT **(B)**, BUN **(C)**, CRE **(C)**, WBCs **(D)**, LYM **(D)**, MON **(D)**, RBCs **(E)**, HGB **(E)**, MCH **(E)**, HCT **(F)**, MCV **(F)**, MCHC **(F)**, PLT **(G)**, PDW **(G)**, MPV **(G)**, RDWs **(H)**, RDWc **(H)**, and PCT **(H)** content in normal mice and ZnONPs-Gel implanted mice at 28 days after SCI. Data are mean ± SD (*n* = 3).

After 28 days of treatment, the toxicity of ZnONPs-Gel to the organs was investigated by histopathological analysis (Figure 8). No obvious change was observed from the H&E-stained major organs of the heart, liver, spleen, lung, and kidney. Moreover, no apparent histopathological abnormalities or lesions were observed in each organ (Figure 8A). The serum biochemistry analysis results (Figures 8B,C) showed that serum concentrations of liver function indicators [aspartate transaminase (AST) and alanine transaminase (ALT)] and kidney function indicators (BUN and CRE) in the ZnONPs-Gel-treated group were similar to those in the normal group (*p* > 0.05), revealing good biocompatibility in the liver and kidney. Moreover, the results of complete blood panel analysis (Figures 8D–H) showed no obvious differences in the hematology of the ZnONPs-Gel USNP-treated group when compared to that of the control group (*p* > 0.05).

## Conclusion

In our study, we conducted a promising therapy for microenvironment regulation based on BMSC-derived ZnONPs adherent on a hydrogel that originated from skeletal muscle. The implantation treatment of ZnONPs-Gel presented high capability in nerve recovery *via* inflammation and ROS inhibition. Taken together, we reported that ZnONPs-Gel application posed a promising ZnONP-loaded implantation approach for an effective and biocompatible treatment of SCI.

## Data Availability

The original contributions presented in the study are included in the article/Supplementary Material, further inquiries can be directed to the corresponding authors.

## References

[B1] Ahuja C. S. Nori S. Tetreault L. Wilson J. Kwon B. Harrop J. (2017). Traumatic Spinal Cord Injury-Repair and Regeneration. Neurosurgery 80, S9–S22. 10.1093/neuros/nyw080 28350947

[B2] Baetke S. C. Lammers T. Kiessling F. (2015). Applications of Nanoparticles for Diagnosis and Therapy of Cancer. Bjr 88, 20150207. 10.1259/bjr.20150207 25969868 PMC4630860

[B3] Basnet P. Inakhunbi Chanu T. Samanta D. Chatterjee S. (2018). A Review on Bio-Synthesized Zinc Oxide Nanoparticles Using Plant Extracts as Reductants and Stabilizing Agents. J. Photochem. Photobiol. B: Biol. 183, 201–221. 10.1016/j.jphotobiol.2018.04.036 29727834

[B4] Basso D. M. Beattie M. S. Bresnahan J. C. (1995). A Sensitive and Reliable Locomotor Rating Scale for Open Field Testing in Rats. J. Neurotrauma 12, 1–21. 10.1089/neu.1995.12.1 7783230

[B5] Boraschi D. Italiani P. Palomba R. Decuzzi P. Duschl A. Fadeel B. (2017). Nanoparticles and Innate Immunity: New Perspectives on Host Defence. Semin. Immunol. 34, 33–51. 10.1016/j.smim.2017.08.013 28869063

[B6] Dadfar S. M. Roemhild K. Drude N. I. von Stillfried S. Knüchel R. Kiessling F. (2019). Iron Oxide Nanoparticles: Diagnostic, Therapeutic and Theranostic Applications. Adv. Drug Deliv. Rev. 138, 302–325. 10.1016/j.addr.2019.01.005 30639256 PMC7115878

[B7] Fawcett J. W. (2015). The Extracellular Matrix in Plasticity and Regeneration after CNS Injury and Neurodegenerative Disease. Prog. Brain Res. 218, 213–226. 10.1016/bs.pbr.2015.02.001 25890139

[B8] Frederickson C. J. Koh J.-Y. Bush A. I. (2005). The Neurobiology of Zinc in Health and Disease. Nat. Rev. Neurosci. 6, 449–462. 10.1038/nrn1671 15891778

[B9] Hernandez M. J. Yakutis G. E. Zelus E. I. Hill R. C. Dzieciatkowska M. Hansen K. C. (2020). Manufacturing Considerations for Producing and Assessing Decellularized Extracellular Matrix Hydrogels. Methods 171, 20–27. 10.1016/j.ymeth.2019.09.015 31546012

[B10] Hickman S. Izzy S. Sen P. Morsett L. El Khoury J. (2018). Microglia in Neurodegeneration. Nat. Neurosci. 21, 1359–1369. 10.1038/s41593-018-0242-x 30258234 PMC6817969

[B11] Hussain I. Singh N. B. Singh A. Singh H. Singh S. C. (2016). Green Synthesis of Nanoparticles and its Potential Application. Biotechnol. Lett. 38, 545–560. 10.1038/s41593-018-0242-x 26721237

[B12] Koffler J. Zhu W. Qu X. Platoshyn O. Dulin J. N. Brock J. (2019). Biomimetic 3D-Printed Scaffolds for Spinal Cord Injury Repair. Nat. Med. 25, 263–269. 10.1038/s41591-018-0296-z 30643285 PMC6559945

[B13] Li H. Ghazanfari R. Zacharaki D. Lim H. C. Scheding S. (2016). Isolation and Characterization of Primary Bone Marrow Mesenchymal Stromal Cells. Ann. N.Y. Acad. Sci. 1370, 109–118. 10.1111/nyas.13102 27270495

[B14] McDonald J. W. Sadowsky C. (2002). Spinal-cord Injury. The Lancet 359, 417–425. 10.1016/s0140-6736(02)07603-1 11844532

[B15] Ogunyemi S. O. Abdallah Y. Zhang M. Fouad H. Hong X. Ibrahim E. (2019). Green Synthesis of Zinc Oxide Nanoparticles Using Different Plant Extracts and Their Antibacterial Activity against Xanthomonas Oryzae Pv. Oryzae. Artif. Cell Nanomedicine, Biotechnol. 47, 341–352. 10.1080/21691401.2018.1557671 30691311

[B16] Pochwat B. Nowak G. Szewczyk B. (2015). Relationship between Zinc (Zn (2+)) and Glutamate Receptors in the Processes Underlying Neurodegeneration. Neural Plast. 2015, 591563. 10.1155/2015/591563 26106488 PMC4461779

[B17] Qin T. Ma R. Yin Y. Miao X. Chen S. Fan K. (2019). Catalytic Inactivation of Influenza Virus by Iron Oxide nanozymeMitochondrial ROS Signaling in Organismal Homeostasis. TheranosticsCell 9163, 6920560–6935569. 10.7150/thno.35826

[B18] Soleimani M. Nadri S. (2009). A Protocol for Isolation and Culture of Mesenchymal Stem Cells from Mouse Bone Marrow. Nat. Protoc. 4, 102–106. 10.1038/nprot.2008.221 19131962

[B19] Ungerleider J. L. Johnson T. D. Rao N. Christman K. L. (2015). Fabrication and Characterization of Injectable Hydrogels Derived from Decellularized Skeletal and Cardiac Muscle. Methods 84, 53–59. 10.1016/j.ymeth.2015.03.024 25843605 PMC4526417

[B20] Zhao X. Wu H. Guo B. Dong R. Qiu Y. Ma P. X. (2017). Antibacterial Anti-oxidant Electroactive Injectable Hydrogel as Self-Healing Wound Dressing with Hemostasis and Adhesiveness for Cutaneous Wound Healing. Biomaterials 122, 34–47. 10.1016/j.biomaterials.2017.01.011 28107663

