# Supplementary material for: Bioengineered Zinc Oxide Nanoparticle-Loaded Hydrogel for Combinative Treatment of Spinal Cord Transection
Source: Front Bioeng Biotechnol. 2022 Jan 13;9:796361. doi: 10.3389/fbioe.2021.796361 (PMC8793849; doi:10.3389/fbioe.2021.796361)
Supplement: Supplementary file 1 [file DataSheet1.PDF]

Bioengineered zinc oxide nanoparticles-loaded hydrogel for combinative treatment of spinal cord transection

Sen Lin[1,#], Hao-sen Zhao[1,#], Chang Xu[1,#], Zi-peng Zhou[1], Da-hao Wang[1], Shu-rui Chen[2,*], Xi-fan Mei[1,*]

[1] Department of Orthopedic, First Affiliated Hospital of Jinzhou Medical University, Jinzhou, P. R. China

[2] Department of Endocrinology, First Affiliated Hospital of Jinzhou Medical University, Jinzhou, P. R. China

[3] Jinzhou Medical University, Jinzhou, P. R. China

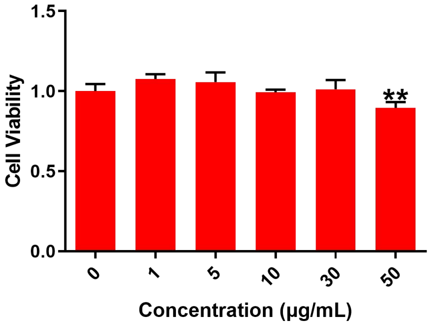

Figure S1. Cell viability of BMSCs treated with ZnONPs.

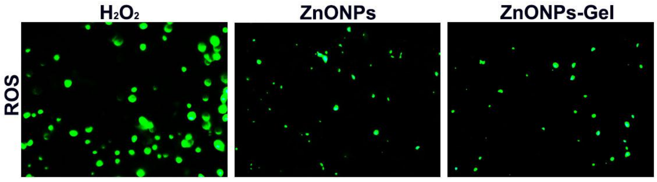

Figure S2. ROS staining of BMSCs treated with various treatments.

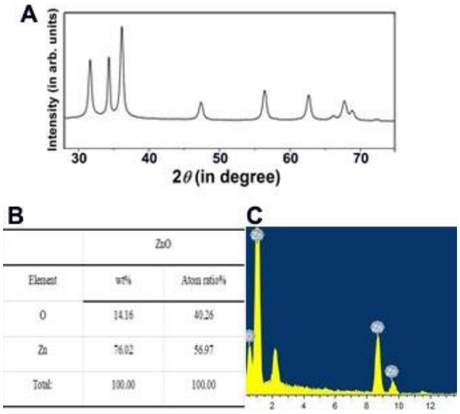

	ZnO	
Element	wt%	Atom ratio%
O	14.16	40.26
Zn	76.02	56.97
Total:	100.00	100.00

Figure S3. XRD and TEM-EDS of ZnO.

The complex vwascosity of resuspension Gel was measured by rheometer in liquid form, and its storage and loss modulus were tested by gel method. The sample was run on a parallel plate rheometer. For complex vwascosity measurements, pipette 200 µL the resuspended liquid gel moves to the center of the parallel plate geometry. The plate temperature was fixed at 25 °C, and the gap height was set to 500 µm. To ensure that the liquid fills the entire gap between the plates. The material runs in a flow program with a programmed frequency of 0.1 to 100 Hz. For storage and loss modulus, the lyophilized gel was resuspended in deionized water and carefully pipetted to 500 µL was transferred to a 4 ml scintillation vial to avoid bubble formation. Gel for 24 hours at 37℃. The sample was run on a plate preset at 37 °C. Carefully transfer the sample to the parallel plate; If the gel breaks, dwascard the gel and use another gel, because any impurity or tear in the gel will affect the mechanical properties of the gel. The gap height was set to 1200 µm, to ensure that the entire gap frequency scanning between gel filled parallel plates and oscillating plates was carried out from 0.1 to 100 rad/s, to measure storage and loss modulus. For thwas size of running gel, the recommended maximum clearance height was 1500µm。

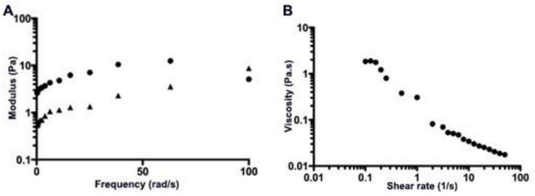

Figure S4. Mechanical and structural properties of ZnONPs-Gel. Rheometer frequency for storage (circular) and loss (triangle) (A). Complex vwascosity for skeletal muscle-derived gel (B).

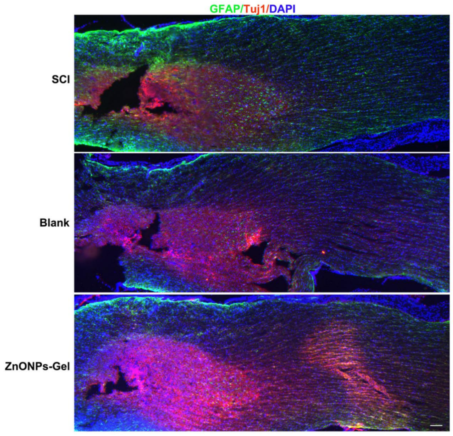

Figure S5. ZnONPs-Gel improved injured spinal cord on days 28 after SCI.

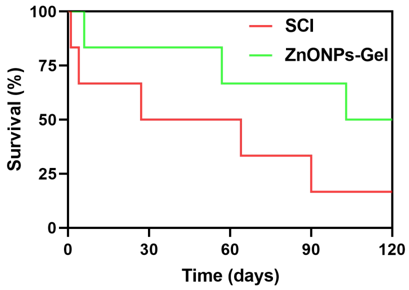

Figure S6. Survival curve of SCI and ZnONPs-Gel groups.
